# Policy Evaluation Network (PEN): Protocol for systematic literature reviews examining the evidence for impact of policies on physical activity across seven different policy domains

**DOI:** 10.12688/hrbopenres.13089.4

**Published:** 2022-01-17

**Authors:** Kevin Volf, Liam Kelly, Enrique García Bengoechea, Bláthín Casey, Anna Gobis, Jeroen Lakerveld, Joanna Zukowska, Peter Gelius, Sven Messing, Sarah Forberger, Catherine Woods, Policy Evaluation Network (PEN) Consortium

**Affiliations:** 1 Physical Activity for Health, Health Research Institute, Department of Physical Education and Sport Sciences, University of Limerick, Limerick, Ireland; 2 Physical Activity for Health Research Cluster, Health Research Institute, University of Limerick, Limerick, Ireland; 3 Faculty of Civil and Environmental Engineering, Gdansk University of Technology, Gdansk, Poland; 4 Amsterdam University Medical Center, Amsterdam, The Netherlands; 5 Friedrich-Alexander-Universität Erlangen-Nürnberg, Erlangen, Germany; 6 Leibniz Institute for Prevention Research and Epidemiology - BIPS, Bremen, Germany

**Keywords:** physical activity, policy, protocol, systematic review, evaluation

## Abstract

**Introduction: **Over 40 million deaths annually are due to noncommunicable diseases, 15 million of these are premature deaths and physical inactivity contributes an estimated 9% to this figure. Global responses have included the Sustainable Development Goals (SDGs) and the Global Action Plan on Physical Activity (GAPPA). Both point to policy action on physical activity (PA) to address change, yet the impact of policy on PA outcomes is unknown.  The protocol described outlines the methodology for systematic literature reviews that will be undertaken by the Policy Evaluation Network (PEN) to address this knowledge gap.

**Methods: **The seven best investments for promotion of population PA identified in the Toronto Charter highlighted seven policy domains (schools, transport, urban design, primary health care systems, public education, community-wide programmes and sport) which will form the basis of these PEN reviews. Seven individual scientific literature searches across six electronic databases will be conducted. Each will use the key concepts of policy, PA, evaluation and a distinct concept for each of the seven policy domains. This will be supplemented with a search of the reference list of included articles. Methodological quality will be assessed and overall effectiveness for each included study will be described according to pre-determined criteria.

**Conclusions: **Each review will provide policy makers with a list of policy statements and corresponding actions which the evidence has determined impact on PA directly or indirectly. By collating the evidence, and demonstrating the depth of the science base which informs these policy recommendations, each review will provide guidance to policymakers to use evidence-based or evidence-informed policies to achieve the 15% relative reduction in physical inactivity as defined by GAPPA.

**Registration: ** PROSPERO CRD42020156630 (10/07/2020).

## Introduction

Physical activity (PA) is defined as “any bodily movement produced by skeletal muscles that requires energy expenditure” ( Caspersen *et al*., 1985). The relationship between PA levels and health outcomes is well established ( Rutten *et al*., 2016). Insufficient PA has been identified by the World Health Organisation (WHO) as the fourth leading risk factor for mortality worldwide ( WHO, 2009) and in 2012 it was estimated that 9% (range 5.1 – 12.5%) of global premature mortality can be attributed to physical inactivity ( Lee *et al*., 2012). The European region has been strongly affected by the costs of inactivity, absorbing 16.9% of the disability that inactivity causes, through its contribution to morbidity from coronary heart disease (CHD), cancer, stroke and diabetes, and 21.8% of the healthcare cost ( Ding *et al*., 2016).

This epidemiological evidence reveals inactivity to be a substantial public health issue and advocacy by public health specialists and the academic community has demanded policy responses to this issue. For the purposes of this document, policy should be understood as “decisions, plans, and actions that are implemented by national or regional governments to achieve specific health promotion goals within a society” ( Lakerveld *et al*., 2020). As indicated by the WHO ( WHO Regional Office for Europe, 2010), policy can give support, coherence and visibility at the political level, while making it possible for the organisations involved at national, regional, and local levels – e.g., national government sectors, regional or local authorities, stakeholders, and the private sector – to be logical and consistent in their actions to achieve a shared goal. This applies to food and PA environments, systems and behaviours ( WHO Regional Office for Europe, 2010). In order to reflect the complexity of the policies that may affect the PA policy environment, Lakerveld & colleagues (2020) distinguish between “direct” policies, which refers to policies where improving the PA environment and increasing participation is the primary aim, and “indirect” policies, where the primary aim of the policy is not to increase PA but this may occur as a co-benefit of successful implementation.

The International Society for Physical Activity and Health (ISPAH) was established in 2009 ( Kohl *et al*., 2012) At its third biennial congress ISPAH promulgated the Toronto Charter calling for political commitment to achieving greater opportunities for PA ( Bull *et al*., 2010). To guide action on this issue the Charter was subsequently accompanied by a document titled Non-Communicable Disease Prevention: Investments that Work for Physical Activity ( ISPAH, 2012). This document declared seven domains which evidence suggested could be effectively targeted to increase PA opportunities. These were whole-of-school programmes, transport policies and systems, urban design regulations and infrastructure, primary health care, public education, community-wide programmes and sport systems and programmes that promote ‘sport for all’. These seven domains provide a policy setting structure for systematic literature review search.

Over recent years there has been an acceleration in the production of policy responses to the epidemics of inactivity and sedentary behaviour ( Klepac Pogrmilovic *et al*., 2018). The Global Observatory for Physical Activity (GoPA) reports that by 2013, 139 countries were members of its PA advocacy alliance and 26.6% of these countries had already published a stand-alone PA plan ( Ramirez Varela *et al*., 2016). Furthermore, in 2013, the WHO published a document which recognised PA as a part of the global effort to combat non-communicable diseases (NCDs) ( WHO, 2013). Another development promoted by the WHO in the field of PA policy is the proliferation of audits of policy responses to inactivity using a tool entitled the Health-Enhancing Physical Activity Policy Audit Tool (HEPA PAT).

A significant development occurred in 2017 when, in response to demands for direction on the problem of physical inactivity, the WHO committed to publishing a stand-alone action plan on this issue. This commitment was realised in 2018 when the WHO published the Global Action Plan on Physical Activity (GAPPA), which targeted an even more ambitious PA target than the previous NCD plan ( WHO, 2018).

The recent rise in the number of national PA policies allows research into the question of which of these policies are effective in improving PA outcomes. A scoping review published in 2016 provided evidence that research into policy effectiveness lagged behind research that links PA to health and research that links PA interventions to behaviour ( Rutten *et al*., 2016). However, with the increase in the number of PA policies there may have been a concomitant rise in research examining the effectiveness of these policies. Furthermore, to the best available knowledge, no project has linked existing policy statements with research that corroborates or discredits the effectiveness of these statements.

As part of the Joint Programming Initiative “A Healthy Diet for a Healthy Life” (JPI HDHL), researchers from 28 institutes in seven European countries (France, Germany, Ireland, Italy, Norway, Poland, and the Netherlands) and New Zealand combine their expertise to form a Policy Evaluation Network (PEN) ( Lakerveld *et al*., 2020; see https://www.jpi-pen.eu/). PEN’s vision is to provide Europe with tools to identify, evaluate and benchmark policies designed to directly or indirectly address unhealthy lifestyle behaviours which contribute to overweight and obesity, while accounting for existing health inequities. Using structured evaluation principles and methods, PEN will examine the content, implementation and impact of lifestyle policies across Europe and will build on existing knowledge. PEN will provide an overview of the ‘best’ public policies most likely to sustainably support more favourable health behaviours.

This protocol paper outlines the methodology for seven complementary systematic literature reviews as part of PEN. Each review is designed to determine the impact of policy, either directly or indirectly, on physical activity outcomes across the different policy domains identified in the “Seven Best Investments” ( ISPAH, 2012). These policy domains are whole-of-school programmes, transport policies and systems, urban design regulations and infrastructure, primary health care, public education, community-wide programmes involving multiple settings and sport systems and programmes that promote ‘sport for all’. These reviews will provide evidence supporting the development of a tool named the Physical Activity Environment Policy Index (PA EPI), based on similar principles to an existing tool called the Food Environment Policy Index (Food EPI) ( Swinburn *et al*., 2013). The PA EPI will provide policy makers with a list of policy statements and corresponding actions which the evidence has determined improve PA outcomes across domains. The aim of each PEN review is to evaluate the status of the evidence base for the impact of policy on PA outcomes across the different policy domains identified in the “seven best investments”.

## Method

Original material examining the evidence of what works in terms of direct and indirect policies to increase PA will be identified in the following ways:

(1) A search, with no date restrictions, of the following electronic databases: four specialized sport science or biomedical databases, MEDLINE (Ebsco), SportDiscus, Cinahl, and Cochrane library, and two general social science databases, Web of Science and Scopus. Search results will be limited to articles that are identified through searching the titles and abstracts.
(2) Manual reference checks of identified original studies.
(3) Publicly available English-language resources and documents of major national and international stakeholders will be searched to identify existing reviews and position papers discussing the evidence of what works in terms of direct and indirect policies for increasing PA, e.g., the WHOs European database on Nutrition, Obesity and Physical Activity (NOPA), Global Action Plan on Physical Activity (GAPPA), the European Physical Activity Strategy (EPAS) ( WHO Regional Office for Europe, 2015) and the European Physical Activity Guidelines (EPAG) ( European Commission, 2008).

A content analysis was performed on the Toronto Charter complementary document ( ISPAH, 2012). These ‘investments’ identified the policy domains or sectors in which policies are made that could directly or indirectly impact on physical activity, i.e., schools, transport, urban design, healthcare, public education, the community and sport. This document was searched for key words to be included in the search syntax. Researchers consulted with librarians and other research staff for suggestions on search terms.

The search of electronic databases will comprise seven individual searches (corresponding to the seven best investments), each one to be run on each of the databases. The seven searches will be formed by combining the same basic search strategy (i.e. general eligibility criteria) with seven distinct search concepts (i.e. specific eligibility criteria for each domain). The basic search strategy will consist of three search concepts ( Table 1): search concept one (C1), which will combine synonyms for the keyword “policy” with the Boolean Operator “OR”; search concept two (C2), which will do the same with the keyword “physical activity”; and search concept three (C3), which will do the same for the keyword “impact”. The three search terms will be combined with the Boolean operator “AND” ( Table 1).

**Table 1.** General Search terms.

Keyword	Synonyms
**“Policy”**	(MH "Policy") OR (MH "Public Policy") OR (MH "Policy Making") OR (“policy”) OR (“policies”) OR (“national policy”) OR (“national framework”) OR (“policy framework”) OR (“policy action”) OR (“legislation”) OR (“strategy”) OR (“policy making”)
**“Physical** **Activity”**	(MH "Exercise") OR (MH “Sedentary Behavior”) OR (“physical activit*”) OR (“physical inactivity”) OR (“play”)OR (“physical education”) OR (“sedentar*”) OR (“sitting”) OR (“healthy lifestyle”) OR (“health initiative”)
**“Impact”**	(“evaluat*”) OR (“impact”) OR (“appraisal”) OR (“effect*”) OR (“assessment”)

Abbreviations: 'MH' = MeSH Heading.

Each of the seven searches will further be combined with a specific search term constructed to reflect only one of the seven best investments declared in the document *Non-Communicable Disease Prevention: Investments that Work for Physical Activity* ( ISPAH, 2012) ( Table 2). It is proposed that individual systematic literature reviews will be performed for each of the seven best investment domains, with an initial review focusing on schools and subsequent reviews focusing on ‘transport’, ‘public education’ and ‘sport’ domains in the first instance.

**Table 2.** Specific Search terms based on each of the seven best investments document ( ISPAH, 2012).

**1. “Whole of School** **Approach”**	“Whole-of-school” OR “Whole School” OR “Whole of School” OR WSCC OR “school intervention” OR “school based intervention” OR “school initiative” OR “school based initiative” OR “school program*” OR “School health” OR “Wellness” OR “well-being”
**2. “Transport** **Policy”**	“active transport*” OR “walk*” OR “cyclist*” OR “bik*” OR “bicycl*” OR “cyclist” OR “cycling” OR "active travel*" OR “commute*” OR "transport mode" OR "transportation mode" OR "travel mode" OR “pedestrian*” OR "traffic volume" OR "traffic count" OR “transport plan*” OR “road safety” OR “public transport” OR “transport systems”
**3. “Urban Design”**	MH “Environment Design” OR MH “Environment” OR MH “Environment and Public Health” OR “urban design” OR “urban environment” OR “built environment” OR “mixed-use development” OR “footpaths” OR “bikeways” OR “street network*” OR “green spac*” OR “green areas” OR “green network” OR “blue spac*” OR “recreational spac*” OR “urban plan*” OR “public amenit*” OR “network infrastructure”
**4. “Primary Health** **care systems”**	“primary health” OR “Primary care” OR “health care” OR “health system”
**5. “Public** **Education”**	“public education” OR “mass media” OR “mass communication” OR “social marketing” OR broadcast* OR MH “Communications Media” OR MH “Social Media” OR “media” OR “health campaigns” OR “public education”
**6. “Community** **Programmes”**	“Whole-of-community” OR “Community-wide programs” “community building” OR “community strengthening” OR "community development" OR "community empowerment" OR "community network*" OR "coalition building" OR "community capacit*" OR "community”
**7. “Sport** **Programmes”**	“health promoting clubs” OR “sport*” OR “athletics”

Abbreviations: 'MH' = MeSH Heading.

The following criteria will be applied for searches in databases: language will be limited to English language only.

### Eligibility criteria

In order to answer our research question some eligibility criteria were developed to screen out irrelevant documents. Studies will be included based on the following criteria for 1) type of study, 2) participants/population, 3) exposure/intervention, and 4) outcomes.

General eligibility criteria were formulated as well as “specific” eligibility criteria for each of the seven searches. Publications that do not meet the “general” eligibility criteria will be excluded from review. Publications that do not meet the “specific” eligibility criteria will be set aside and possibly reassigned to a different search category if they are not duplicates of any publication already included in that search category.

***Types of study to be included/excluded.*** No limitations regarding study type will be placed as long as the study design allows the research questions to be addressed. In addition, reviews using a comprehensive search strategy (including systematic, scoping and realist reviews) and analysing original research on the evidence of what works, in terms of direct and indirect policies for increasing PA; and reviews and policy analysis documents issued by major national and international organisations addressing recommendations referring to the same evidence will be eligible for inclusion. Studies will be excluded based on the following criteria: a direct or indirect form of policy intervention is not identifiable; no information is provided regarding the effects of the policy under consideration on the desired outcomes.

***Condition or domain being studied.*** Reviews examining the evidence of what works in terms of direct and indirect policies on PA.

***Participants/population.*** Eligibility criteria relating to population characteristics are described in Table 3.

**Table 3.** Population related inclusion criteria.

General criteria	School specific criteria	Transport specific criteria	Urban design specific criteria	Primary health care specific criteria	Public education specific criteria	Community programmes specific criteria	Sport programmes specific criteria
The study intervention targets the **general** **human** **population** or parts of it that are relevant for the respective review	The study intervention targets **students** **and staff** in the **school** **setting.**	The study intervention targets the **commuters** and their preferred mode **of** **transport.**	The study intervention targets the **residents** **of urban** **areas**	The study intervention targets **patients** or **primary care** **professionals**	The study intervention targets the **general** **population** through public outreach and mass communication.	The study intervention targets the **general** **population** in the community setting.	The study intervention targets the **general** **population** in **sport** **settings.**

***Exposure(s), intervention(s).*** Policies that aim to have a direct or indirect effect on PA behaviour of target groups and populations and on the PA environment that support the behaviour under consideration.

Grey literature/Other: Similar to the empirical studies, included grey literature will need to make reference to the impact of PA policy in the relevant domain.

***Context.*** These systematic reviews are performed as a task of PEN. PEN’s vision is to provide Europe with tools to identify, evaluate and benchmark policies designed to directly or indirectly address physical inactivity. Further information on PEN is available at www.jpi-pen.eu or Lakerveld & colleagues (2020)

***Main outcome(s).*** All study designs (e.g., reviews, empirical evidence) and grey literature/other must include the following outcome(s); a changes in PA (or proxy, e.g. fitness), assessed by means of self-report or wearable devices (e.g., accelerometer); a change in features of the physical and social environment (e.g., facilities, equipment, action plans, programmes) hypothesised to lead to changes in PA outcomes as a result of a policy intervention.

### Study selection and data extraction

***Download of title and abstract records.*** Titles and abstracts identified by the search will be downloaded as “Endnote import” (extension.enw) files or other file formats compatible with our software. They will be uploaded to Endnote X9, a citation management software, and Rayyan ( Ouzzani *et al*., 2016), an online software dedicated to managing reviews. Other freely available alternative software includes Mendeley reference manager or Zotero. Once records have been uploaded to Rayyan, the software will identify duplicate articles and one of the two identical articles will be removed. The remaining articles will undergo the first round of screening by two researchers in a shared Rayyan account.

***Title and abstract review.*** Title and abstract reviews will be performed by at least one reviewer and checked by another reviewer. Checking will involve reviewing title and abstracts decisions to establish whether the second reviewer concurs with the screening decision. Discrepancies will be resolved by discussion to reach consensus, in consultation with a third researcher when necessary. The screening process will involve comparing the information presented in the title and abstract to the eligibility criteria. Titles and abstracts that appear to conform to the eligibility criteria will be deemed eligible for full text review while those that do not will be discarded from the next stage of the data extraction process.

***Download of full articles.*** Full text articles will be downloaded using the resources provided by their Institution. If a full text record cannot be acquired using these resources, researchers will investigate whether they can be located through use of other libraries to which the research team has access. If a full text article cannot be located through any of these library resources, the authors will be contacted through whichever channels can be identified from the information in the title and abstract.

***Full text review.*** Full text reviews will be performed by at least one reviewer and checked by a second reviewer. The authors involved in screening and risk of bias will differ between the different reviews; however, the authors involved in full text screening will be the same authors with responsibility for title and abstract screening. Discrepancies will be resolved by discussion to reach consensus, in consultation with a third researcher when necessary. The following information will be extracted: first author, year of publication, country, study design, data collection method, sample, recruitment/setting, sample size, and response rate.

### Risk of bias (quality) assessment

Risk of bias will be assessed by at least one reviewer and checked by another reviewer. Discrepancies will be resolved by discussion to reach consensus, in consultation with a third researcher when necessary. The results of the quality assessment will be narratively incorporated into the synthesis process. A descriptive summary using the criteria described below will be presented at study level and discussed in the review. Furthermore, the methodological quality will be narratively summarized at review level.

The quality of the included quantitative studies, inclusive of randomised, non-randomised and observational studies (encompassing both longitudinal and cross-sectional studies) will be assessed by means of an adapted ‘Downs and Black’ checklist tool ( Downs & Black, 1998). This tool is apt to assess common biases in a range of study types as noted. The checklist will be modified to meet the aims of this review with some items deemed non applicable and subsequently removed.

The AMSTAR tool will be used for the assessment of systematic reviews and comprehensive reviews with a rigorous search strategy including reviews of reviews. This tool consists of 11 items and has good face and content validity for appraising the methodological quality of systematic reviews ( Shea *et al*., 2007). Not all items are applicable to every type of review being assessed and quality ratings will take account of this circumstance. Similar to Messing *et al*. (2019), to assess the quality of included studies, we will calculate percentage values for each study. Each study will be assessed by a tool appropriate for its study design and these percentage values will be calculated based on the percentages of criteria met by a study, which will be particular to the tool used to assess it.

### Strategy for data synthesis

A narrative synthesis will be used to interpret and analyse the data. The results of the data synthesis will be presented in a table. Within this table, a list of short descriptive statements will be compiled based on the policy actions identified in the scientific literature. Evidence on the effectiveness of these policy actions will then be described using a method used by Panter & colleagues (2019). Specifically, when a study presents quantitative evidence about the effectiveness of one policy action a symbol will be assigned next to that particular policy action in the table. There are four categories of symbols reflecting the four possible outcomes: “significantly positive evidence” (+), “significantly negative evidence” (-), “no significance test” (?) or “inconclusive” (0). In summary, the data synthesis table will display three types of data: brief policy statements, codes which help visualise the number and direction of effects found in the literature supporting those statements, and the supporting references. In addition, data extraction tables will be designed to distinguish any demographic, environmental or other variables pertinent to synthesising the data. For example, in the schools’ review, data extraction columns will be included to reflect evidence of effectiveness stratified by gender, school level (primary, secondary, combined) or socio-economic status where appropriate. 

Finally, for the included reviews and policy analysis documents, the main findings stated in the discussion and conclusions section will be extracted. Main findings of the articles will be copied into a single table along with a reference to the article itself, and details of the overall risk of bias of the study from which the information is extracted. The synthesised data will be presented in a six-column table with the different columns presenting information on the reference, study description, study type, main findings or outcomes, risk of bias and category of evidence, respectively.

### Dissemination

Study findings will be presented at professional networking events such as the World Congress on Public Health. Manuscripts will be prepared for publication in scientific peer-reviewed journals and presented at academic conferences.

### Study status

The submission of the first of seven intended reviews is being finalised, this focuses on the school setting A further three reviews are underway, these will focus on transport, public education and sport policy domains.

## Conclusion

An aim of this project is to assist policymakers to achieve the GAPPA target of a 15% relative reduction in the prevalence of insufficient PA ( WHO, 2018). The aim of the planned work is to determine the level and type of evidence reported in the international scientific literature for policies that contribute directly or indirectly to increasing PA within 7 priority domains identified as best investments for PA ( ISPAH, 2012). This will be achieved through searches electronic databases and extensive snowballing techniques. By providing this evidence, these reviews will support the development of the PA-EPI. The PA-EPI in turn will support policy makers by facilitating the benchmarking of policies which work towards achieving this target. Achieving this target will provide health benefits such as reduced premature mortality as well as substantial co-benefits such as contributing to a sustainable environment and quality education ( WHO, 2018). We anticipate that the recommendations will mirror and expand upon some of the prescriptions made in GAPPA, the European Physical Activity Strategy or by experts in the PA community

## Data availability

### Underlying data

No underlying data are associated with this article.

### Reporting guidelines

Open Science Framework: PRISMA-P checklist for “Policy Evaluation Network (PEN): Protocol for systematic literature review examining the evidence for impact of school policies on physical activity”. https://doi.org/10.17605/OSF.IO/26QYF ( Volf, 2020).

Data are available under the terms of the Creative Commons Zero "No rights reserved" data waiver (CC0 1.0 Public domain dedication).
