# Supplementary material for: Policy Evaluation Network (PEN): Protocol for systematic literature reviews examining the evidence for impact of policies on physical activity across seven different policy domains
Source: HRB Open Res. 2022 Jan 17;3:62. Originally published 2020 Sep 4. [Version 4] doi: 10.12688/hrbopenres.13089.4 (PMC8567685; doi:10.12688/hrbopenres.13089.4)
Supplement: Peer review history

# Peer review: Reviewer response for version 4

**DOI:** 10.21956/hrbopenres.14693.r31259

**Published:** 2022-01-26

**Authors:** Eric Breton

**Affiliations:** 1 Social and Human Sciences Department, École des hautes études en santé publique, Rennes, France; 2 Arènes research unit (UMR CNRS 6051: team INSERM U1309), Rennes, France

I am satisfied with the answers given to the points I raised in my first report.

Is the study design appropriate for the research question?

Yes

Is the rationale for, and objectives of, the study clearly described?

Yes

Are sufficient details of the methods provided to allow replication by others?

Yes

Are the datasets clearly presented in a useable and accessible format?

Yes

Reviewer Expertise:

Community health, prevention strategies, health promotion, capacity building strategies for the prevention of chronic diseases.

I confirm that I have read this submission and believe that I have an appropriate level of expertise to confirm that it is of an acceptable scientific standard.

# Peer review: Reviewer response for version 3

**DOI:** 10.21956/hrbopenres.14427.r30448

**Published:** 2021-11-17

**Authors:** Eric Breton

**Affiliations:** 1 Social and Human Sciences Department, École des hautes études en santé publique, Rennes, France; 2 Arènes research unit (UMR CNRS 6051: team INSERM U1309), Rennes, France

Dear authors,

 Informing policy-makers on the best options available to improve the level of physical activity across whole populations is definitely the next step after years of research looking at the determinants of physical activity. This project is therefore likely to generate valuable evidence that will not only pinpoint the best policies but also facilitate advocacy for policy change.

 I was surprised not seeing any reference to the Physical Activity Policy Analysis Tool (HEPA PAT) that allows for a diagnosis of national policy responses to physical inactivity. I think it should be mentioned as part of the current efforts to improve policies.

 The general methods is appropriate but is likely going to be adapted as work on the 7 domains will be carried out. Other search and inclusion/exclusion criteria are likely to be stated when facing the large body of papers published in some of the domains.

 In the following lines, I address some of the specific issues I identified.

- Second paragraph of the introduction: the last sentence seems at odd with the topic covered by the paragraph (“The International Society for Physical Activity and Health (ISPAH) was established in 2009 (Kohl *et al.*, 2012) and numerous articles and editorials in leading academic and medical journals have pointed out the need to address physical inactivity (Bull & Bauman, 2011; Das & Horton, 2012; Kohl *et al*., 2012; Woods & Mutrie, 2012).”) - It is not fitting well there.
- In the fourth paragraph, you wrote: “Over recent years there has been an acceleration in the production of policy responses to the epidemics of physical activity and sedentary behaviour” - Seems odd talking about the epidemics of physical activity.
- In table 1 on the search terms for policy: there is a fair bit of overlap. Searching “policy” as a single term will automatically yield hits that include “national policy”. Therefore no need to enter the latter. I assume the terms that will not be searched in the MH will be searched in the title/abstract as mentioned in the text. In the “impact” search string, shouldn’t “outcome” be included?
- In Table 2: in the “transport policy” search string, “cyclist” appears twice and “Commute*” should be entered as “commut*”.
- In the “urban design” one, “urban environment” appears twice as well. If I am not wrong in the literature, there are mentions along the green spaces to the blue spaces. Lakes and waterways can be settings for physical activity.
- For the “Primary Health care systems” search string, I would add “primary care”.
- For the “Community Programmes” string, I do think the term “capacity building” will generate many hits that are not relevant. I would remove it and add “community building” and “community strengthening”.
- In table 3, under the “Urban design specific criteria” we read that: “The study intervention targets the residents of urban areas”. I did not understand that this idea of using the leverage of actions on urban design was only for urban areas. If that is the case, it would definitely leave behind a significant share of populations that are known to present a poorer health status.

This is all I can see reading this paper.

Is the study design appropriate for the research question?

Yes

Is the rationale for, and objectives of, the study clearly described?

Yes

Are sufficient details of the methods provided to allow replication by others?

Yes

Are the datasets clearly presented in a useable and accessible format?

Yes

Reviewer Expertise:

Community health, prevention strategies, health promotion, capacity building strategies for the prevention of chronic diseases.

I confirm that I have read this submission and believe that I have an appropriate level of expertise to confirm that it is of an acceptable scientific standard, however I have significant reservations, as outlined above.

# Response

**Published:** 2021-12-07

**Authors:** Kevin Volf

**Affiliations:** University of Limerick, Ireland

Dear Dr Breton,

 Thank you for your feedback on our paper. By our reading, you have highlighted 9 different points for improvement in the paper. We quote each point and provide our response below.

- “I was surprised not seeing any reference to the Physical Activity Policy Analysis Tool (HEPA PAT) that allows for a diagnosis of national policy responses to physical inactivity. I think it should be mentioned as part of the current efforts to improve policies.”

The following sentence has been added to the introduction (lines 45 to 47) to add reference to the HEPA PAT: “Another development promoted by the WHO in the field of PA policy is the proliferation of audits of policy responses to inactivity using a tool entitled the Health-Enhancing Physical Activity Policy Audit Tool (HEPA PAT).”

- “Second paragraph of the introduction: the last sentence seems at odd with the topic covered by the paragraph (“The International Society for Physical Activity and Health (ISPAH) was established in 2009 (Kohl et al., 2012) and numerous articles and editorials in leading academic and medical journals have pointed out the need to address physical inactivity (Bull & Bauman, 2011; Das & Horton, 2012; Kohl et al., 2012; Woods & Mutrie, 2012).”) - It is not fitting well there.”

We agree that this seems out of place. We have moved the first clause and deleted the second, this influences the reference list which we have also updated accordingly. Paragraph two (lines 12 to 27) now reads: “This epidemiological evidence reveals inactivity to be a substantial public health issue and advocacy by public health specialists and the academic community has demanded policy responses to this issue. For the purposes of this document, policy should be understood as “decisions, plans, and actions that are implemented by national or regional governments to achieve specific health promotion goals within a society” ( Lakerveld *et al*., 2020). As indicated by the WHO ( WHO Regional Office for Europe, 2010), policy can give support, coherence and visibility at the political level, while making it possible for the organisations involved at national, regional, and local levels – e.g., national government sectors, regional or local authorities, stakeholders, and the private sector – to be logical and consistent in their actions to achieve a shared goal. This applies to food and PA environments, systems and behaviours ( WHO Regional Office for Europe, 2010). In order to reflect the complexity of the policies that may affect the PA policy environment, Lakerveld & colleagues (2020) distinguish between “direct” policies, which refers to policies where improving the PA environment and increasing participation is the primary aim, and “indirect” policies, where the primary aim of the policy is not to increase PA but this may occur as a co-benefit of successful implementation.”

- “In the fourth paragraph, you wrote: “Over recent years there has been an acceleration in the production of policy responses to the epidemics of physical activity and sedentary behaviour” - Seems odd talking about the epidemics of physical activity.”

We have amended this sentence to read "...epidemics of *inactivity* and sedentary behaviour” [line 40].

- “In table 1 on the search terms for policy: there is a fair bit of overlap. Searching “policy” as a single term will automatically yield hits that include “national policy”. Therefore, no need to enter the latter. I assume the terms that will not be searched in the MH will be searched in the title/abstract as mentioned in the text. In the “impact” search string, shouldn’t “outcome” be included?”

The assumption that terms that are not searched in the MH will be searched by title and abstract is correct. As some of the reviews have been commenced and submitted, we cannot amend the general search string. However, other suggestions made below have been incorporated.

- “In Table 2: in the “transport policy” search string, “cyclist” appears twice and “Commute*” should be entered as “commut*”.”

Since the transport review has been completed and is currently under review by an academic journal, the transport policy search string cannot be revised.

- “In the “urban design” one, “urban environment” appears twice as well. If I am not wrong in the literature, there are mentions along the green spaces to the blue spaces. Lakes and waterways can be settings for physical activity.”

We have amended the search terms so that "Blue spac*" is added, and the second "urban environment" removed in the urban design search string.

- “For the “Primary Health care systems” search string, I would add “primary care”.”

We have added the search term "primary care *" to the primary health care search string.

- “For the “Community Programmes” string, I do think the term “capacity building” will generate many hits that are not relevant. I would remove it and add “community building” and “community strengthening”.”

We have amended the search terms so that “community building” and “community strengthening” are added, and “community strengthening” is removed from the community programmes search string.

- “In table 3, under the “Urban design specific criteria” we read that: “The study intervention targets the residents of urban areas”. I did not understand that this idea of using the leverage of actions on urban design was only for urban areas. If that is the case, it would definitely leave behind a significant share of populations that are known to present a poorer health status.”

It is correct that the urban design review will not reach rural populations. It is expected that policy actions with evidence for effectiveness will include actions such as mixed urban land use. However, rural communities will be reached through the other reviews.

 Kind regards,

 Kevin Volf

# Peer review: Reviewer response for version 3

**DOI:** 10.21956/hrbopenres.14427.r30447

**Published:** 2021-11-03

**Authors:** Petru Sandu

**Affiliations:** 1 Faculty of Political, Administrative and Communication Sciences, Department of Public Health, Babes-Bolyai University, Cluj-Napoca, Romania

This article presents very clearly the process undertaken for conducting 7 SLRs looking at the impact of PA policies in 7 priority settings towards established PA related outcomes. 

 The introduction is very clear and comprehensive, gives a good overview of the research in this area and the need for the current project.

 The methods are also clear, concise but comprehensive. I only have one question: the two researchers conducting title and abstract, full-text review and risk of bias assessment are the same to researchers of there are 3 sets of 2 researchers? It is not obvious from the text. Also, who is the 3r researcher in each case? I guess details about this will be described in the following papers, but I think something should be also mentioned in this paper. 

 The strategy for data synthesis is also clear but I think the specific data included in the tables for each of the 7 reviews should be more clearly defined. Again, probably these will be detailed in future papers. 

 The conclusion seems more like a impact paragraph to me. It is necessary and useful, but not as conclusions. Conclusions should refer to the study aim, methods put in place and envisioned results.

 Also, some phrasing related feedback:

1. *Over recent years there has been an acceleration in the production of policy responses to the epidemics of physical activity and sedentary behaviour* - do you mean physical inactivity and sedentary behaviour?
2. Instead of: *Original material examining the evidence of what works in terms of direct and indirect policies on PA will be identified in the following ways - *Original material examining the evidence of what works in terms of direct and indirect policies to increase PA will be identified in the following ways.

In conclusion, I think this paper is a good description of the methods put in place by PEN network in order to conduct a series of SLRs aimed at exploring the impact of PA policy on selected PA related outcome, with the final aim to assist decision makers in making evidence informed investments.

Is the study design appropriate for the research question?

Yes

Is the rationale for, and objectives of, the study clearly described?

Yes

Are sufficient details of the methods provided to allow replication by others?

Yes

Are the datasets clearly presented in a useable and accessible format?

Not applicable

Reviewer Expertise:

Physical activity policy

I confirm that I have read this submission and believe that I have an appropriate level of expertise to confirm that it is of an acceptable scientific standard.

# Response

**Published:** 2021-12-07

**Authors:** Kevin Volf

**Affiliations:** University of Limerick, Ireland

Dear Dr Sandu,

 Thank you for your feedback on our review protocol. This will contribute significantly to the improvement of this paper. By our reading, you have highlighted 5 different points for improvement in the paper. We quote each point and provide our response below.

- “The methods are also clear, concise but comprehensive. I only have one question: the two researchers conducting title and abstract, full-text review and risk of bias assessment are the same to researchers of there are 3 sets of 2 researchers? It is not obvious from the text. Also, who is the 3r researcher in each case? I guess details about this will be described in the following papers, but I think something should be also mentioned in this paper.”

As there are several reviews planned, and responsibility for these tasks will differ between reviews, it is not possible to assign authors initials to each of the roles specified in this publication. However, we have added the following to the paragraph entitled ‘full text reviews’ to provide greater clarity: “The authors involved in screening and risk of bias will differ between the different reviews; however, the authors involved in full text screening will be the same authors with responsibility for title and abstract screening” [lines 193 to 195].

- The strategy for data synthesis is also clear but I think the specific data included in the tables for each of the 7 reviews should be more clearly defined. Again, probably these will be detailed in future papers. “To provide further clarity on how the data synthesis table can be visualised the following was added to the second paragraph of the subsection entitled ‘Strategy for data synthesis’: “In summary, the data synthesis table will display three types of data: brief policy statements, codes which help visualise the number and direction of effects found in the literature supporting those statements, and the supporting references” [lines 230 to 233].

The data extraction tables will display descriptive data on the papers including general data in of the papers (first author, date, country) descriptions of the population (sample size, demographics), description of the policy itself and reported effects.

- “The conclusion seems more like a impact paragraph to me. It is necessary and useful, but not as conclusions. Conclusions should refer to the study aim, methods put in place and envisioned results.”

To provide reference to the study aim the following sentence has been added: “The aim of the planned work is to determine the level and type of evidence reported in the international scientific literature for policies that contribute directly or indirectly to increasing PA within 7 priority domains identified as best investments for PA” [lines 255 to 258].

 To refer to the methods the following line has been added “this will be achieved through searches electronic databases and extensive snowballing techniques” [lines 258 to 259].

 To address the lack of envisioned results the following sentence has been added to the conclusion: “We anticipate that the recommendations will mirror and expand upon some of the prescriptions made in GAPPA, the European Physical Activity Strategy or by experts in the PA community.” [lines 264 to 266].

- “Over recent years there has been an acceleration in the production of policy responses to the epidemics of physical activity and sedentary behaviour - do you mean physical inactivity and sedentary behaviour?”

We have amended this sentence to read "...epidemics of *inactivity* and sedentary behaviour” [line 40].

- “Instead of: Original material examining the evidence of what works in terms of direct and indirect policies on PA will be identified in the following ways - Original material examining the evidence of what works in terms of direct and indirect policies to increase PA will be identified in the following ways.”

We have amended this sentence so that "On PA" is replaced with "to increase PA" [line 88].

 Kind regards

 Kevin Volf

# Peer review: Reviewer response for version 3

**DOI:** 10.21956/hrbopenres.14427.r29226

**Published:** 2021-03-31

**Authors:** Melinda Craike

**Affiliations:** 1 Institute for Health and Sport, Victoria University, Melbourne, Vic, Australia; 2 Mitchell Institute, Victoria University, Melbourne, Australia

No new comments.

Is the study design appropriate for the research question?

Partly

Is the rationale for, and objectives of, the study clearly described?

Partly

Are sufficient details of the methods provided to allow replication by others?

Partly

Are the datasets clearly presented in a useable and accessible format?

Not applicable

Reviewer Expertise:

Physical activity behaviour change; systematic reviews.

I confirm that I have read this submission and believe that I have an appropriate level of expertise to confirm that it is of an acceptable scientific standard.

# Peer review: Reviewer response for version 2

**DOI:** 10.21956/hrbopenres.14323.r28482

**Published:** 2020-12-17

**Authors:** Melinda Craike

**Affiliations:** 1 Institute for Health and Sport, Victoria University, Melbourne, Vic, Australia; 2 Mitchell Institute, Victoria University, Melbourne, Australia

I thank the Authors for their thorough responses to my comments and proposed revisions. I am happy with the revisions and have no further comments. I wish the Authors all the best with this series of reviews.

Is the study design appropriate for the research question?

Partly

Is the rationale for, and objectives of, the study clearly described?

Partly

Are sufficient details of the methods provided to allow replication by others?

Partly

Are the datasets clearly presented in a useable and accessible format?

Not applicable

Reviewer Expertise:

Physical activity behaviour change; systematic reviews.

I confirm that I have read this submission and believe that I have an appropriate level of expertise to confirm that it is of an acceptable scientific standard.

# Peer review: Reviewer response for version 1

**DOI:** 10.21956/hrbopenres.14190.r28217

**Published:** 2020-11-05

**Authors:** Melinda Craike

**Affiliations:** 1 Institute for Health and Sport, Victoria University, Melbourne, Vic, Australia; 2 Mitchell Institute, Victoria University, Melbourne, Australia

Thank you for the invitation to review this paper. This protocol paper outlines the details of the first of several planned systematic literature reviews to assess the impact of policy on physical activity outcomes based on the “seven best investments” document (ISPAH, 2012). This is a very ambitious review series and I commend the authors on undertaking this important work. While the methods to be employed is generally clear, there are several areas that require more detailed explanation/rationale. I believe that doing so will strengthen this paper and ultimately, the review.

Introduction

 It would be helpful to include specific aims of the systematic review.

P 4- Eligibility criteria

 The authors need to clearly define what they mean by a ‘whole of school approach’ what are the critical components or does a policy just need to be labelled as such? For examples, does a ‘whole of school’ approach need to address the elements identified in ISPAH 2012: i.e. “prioritizing: regular, highly-active, physical education classes; providing suitable physical environments and resources to support structured and unstructured physical activity throughout the day (e.g., play and recreation before, during and after school); supporting walk/cycle-to-school programs and enabling all of these actions through supportive school policy and engaging staff, students, parents and the wider community”.

 I was confused by the distinction between the “mandatory” eligibility criteria and specific “preferred” eligibility criteria and the rationale for this. I have not seen this before in a review. Can authors more clearly explain the application of these and the rationale? Perhaps by way of examples?

p. 5 Main Outcomes

 The Introduction section implies that the focus of the review will be on the effectiveness of policies in increasing PA. This seems to be inconsistent with the Main Outcome(s), identified on p. 8, where studies will be eligible if they include “a change in features of the physical and social environment (e.g., facilities, equipment, action plans, programmes) hypothesised to lead to changes in PA outcomes as a result of a policy intervention”. I suggest that the Introduction include a section that alludes to this, and why such outcomes could be considered important (e.g., that focusing on mediators can be used as a proxy for actional PA participation outcomes).

p. 6 Full text review

 Given the importance of implementation to the success of policies, I wonder if it would be worthwhile for the authors to attempt to gather data to indicate whether or not the policy was implemented? Or whether, for example, action plans were developed, and resources allocated to implementation. Given the importance of implementation to policy success, I would see this type of information as critical when evaluating the success of such policies.

 Also, the inclusion of the validity and reliability of the PA measures should be included in the data extraction.

 Each of the policy types is very broad, which will make it difficult to identify policy action that is needed to increase PA. For example, a ‘whole-of-school approach’ can includes several different actions. Have the authors considered providing more detail or categorising the type of whole of school approach to facilitate a clearer and more meaningful assessment about the characteristics of effective whole of school policies?

p. 6. Strategy for data synthesis

 I am not clear on what is meant by: “Outcome data extracted will be tabulated to determine the impact **on** ( *my emphasis*) policy areas and policy actions (policy indicators)”. What is meant here by ‘policy indicators’ for example?

 I suggest that the method described by Panter and colleagues (2019) be described in the paper (rather than just referring the reader to the paper). It is very important that the reader understands the criteria that is being used to assess the evidence of effectiveness.

 I am unclear about how the findings from systematic reviews could be combined with primary research studies. This needs to be further explained.

 Will there be any attempt to distinguish effectiveness based on the following: school level (primary or secondary school level), gender, level of socio-economic disadvantage?

Is the study design appropriate for the research question?

Partly

Is the rationale for, and objectives of, the study clearly described?

Partly

Are sufficient details of the methods provided to allow replication by others?

Partly

Are the datasets clearly presented in a useable and accessible format?

Not applicable

Reviewer Expertise:

Physical activity behaviour change; systematic reviews.

I confirm that I have read this submission and believe that I have an appropriate level of expertise to confirm that it is of an acceptable scientific standard, however I have significant reservations, as outlined above.

# Response

**Published:** 2020-11-18

**Authors:** Kevin Volf

**Affiliations:** University of Limerick, Ireland

Dear Dr Craike,

 On behalf of the review team, I would like to thank you for taking the time for your thorough review of our paper. The team would especially like to thank you for the timely manner in which your review was conducted. We have received your feedback and have made changes to the manuscript accordingly, and believe this has strengthened the quality of the paper.

 Please find below a point-by-point response to the issues raised in your review, with changes from the previous version highlighted in red, for your consideration.

 Yours sincerely,

 Kevin Volf, B.Sc., MM.Sc. PhD Candidate

**Comment 1:**

Introduction

 It would be helpful to include specific aims of the systematic review.

 --

 We agree with the comment and the aims have added to the final paragraph of the Introduction section. To clarify the focus of this paper is to provide information on the protocol being used for seven reviews, this was unclear in the original manuscript.  The text has been edited to make this point clearer throughout. Please see amendments below, text in bold is ‘new’ and has been added for clarity:

 [Introduction page 6, paragraph 2]

 At its third biennial congress ISPAH promulgated the Toronto Charter calling for political commitment to achieving greater opportunities for PA (Bull et al., 2010). To guide action on this issue the Charter was subsequently accompanied by a document titled Non-Communicable Disease Prevention: Investments that Work for Physical Activity (2012). This document declared seven domains which evidence suggested could be effectively targeted to increase PA opportunities. **These were whole-of-school programmes, transport policies and systems, urban design regulations and infrastructure, primary health care, public education, community-wide programmes and sport systems and programmes that promote ‘sport for all’.** These seven domains provide a **policy setting** structure for systematic literature review search.

 [Introduction page 7 paragraph 1]

 This protocol paper outlines the methodology for seven complementary systematic literature reviews **as part of PEN. Each review is** designed to determine the impact of policy **, either directly or indirectly,** on physical activity outcomes **across** the different policy domains identified in the “Seven Best Investments” ( ISPAH, 2011). **These policy domains are schools, transport, urban design, healthcare, public education, the community and sport.** **These** reviews will provide evidence supporting the development of a tool named the Physical Activity Environment Policy Index (PA EPI), based on similar principles to the Food Environment Policy Index (Food EPI) ( Swinburn *et al*., 2013). The PA EPI will provide policy makers with a list of policy statements and corresponding actions which the evidence has determined improve PA outcomes across domains. **The aim of each PEN review is to evaluate the status of the evidence base for the impact of policy on PA outcomes across the different policy domains identified in the “seven best investments”.**

**Comment 2:**

 P 4- Eligibility criteria

 The authors need to clearly define what they mean by a ‘whole of school approach’ what are the critical components or does a policy just need to be labelled as such? For examples, does a ‘whole of school’ approach need to address the elements identified in ISPAH 2012: i.e. “prioritizing: regular, highly-active, physical education classes; providing suitable physical environments and resources to support structured and unstructured physical activity throughout the day (e.g., play and recreation before, during and after school); supporting walk/cycle-to-school programs and enabling all of these actions through supportive school policy and engaging staff, students, parents and the wider community”.

 --

 The reference to ‘whole of school approach’ is directly related to the ‘Seven Investments’ paper.  The use of ‘Whole of school’ within the search strategy as part of the schools domain, is not for the purpose of evaluating the impact of ‘whole of school programmes’, rather to review policies pertinent to the school setting, to determine if any evidence of policy impact exists.  The text has been modified as follows, text in bold is ‘new’ and has been added for clarity;

 [Introduction page 7 paragraph 1]

 This protocol paper outlines the methodology for seven complementary systematic literature reviews as part of PEN. Each review is designed to determine the impact of policy, either directly or indirectly, on physical activity outcomes across the different policy domains identified in the “Seven Best Investments” ( ISPAH, 2011). **These policy domains are whole-of-school programmes, transport policies and systems, urban design regulations and infrastructure, primary health care, public education, community-wide programmes involving multiple settings and sport systems and programmes that promote ‘sport for all’**

 It is proposed that individual systematic literature reviews will be performed on each of the seven best investment domains, with this initial review focusing on schools and subsequent reviews focusing on ‘transport’, ‘public education’ and ‘sport’ domains.”

**Comment 3:**

 I was confused by the distinction between the “mandatory” eligibility criteria and “preferred” eligibility criteria and the rationale for this. I have not seen this before in a review. Can authors more clearly explain the application of these and the rationale? Perhaps by way of examples?

 --

  In essence, the “mandatory” and “preferred” nomenclature refer to “elements that are common to all reviews” and “elements that only relate to a particular review” respectively. While this was explained in the search details, to make this clearer we have edited the mandatory and preferred labels to “general” (applies to all mooted reviews) and “specific” (only applies to a particular review) as follows, text in bold is ‘new’ and has been added for clarity:

 [Methods, p7 paragraph 5]:

 “The search of electronic databases will comprise seven individual searches (corresponding to the seven best investments), each one to be run on each of the databases. The seven searches will be formed by combining the same basic search strategy **(i.e., general eligibility criteria)** with seven distinct search concepts **(i.e., specific eligibility criteria for each domain).  **The basic search strategy will consist of three search concepts ( Table 1): search concept one (C1), which will combine synonyms for the keyword “policy” with the Boolean Operator “OR”; search concept two (C2), which will do the same with the keyword “physical activity”; and search concept three (C3), which will do the same for the keyword “impact”. The three search terms will be combined with the Boolean operator “AND” ( Table 1).

 Each of the seven searches will further be combined with a specific search concept constructed to reflect only one of the seven best investments declared in the document *Non-Communicable Disease Prevention: Investments that Work for Physical Activity (* *ISPAH, 2011* *)* ( Table 2). It is proposed that individual systematic literature reviews will be performed for each of the seven best investment domains, with an initial review focusing on schools and subsequent reviews focusing on ‘transport’, ‘public education’ and ‘sport’ domains.

**Comment 4:**

 p. 5 Main Outcomes

 The Introduction section implies that the focus of the review will be on the effectiveness of policies in increasing PA. This seems to be inconsistent with the Main Outcome(s), identified on p. 8, where studies will be eligible if they include “a change in features of the physical and social environment (e.g., facilities, equipment, action plans, programmes) hypothesised to lead to changes in PA outcomes as a result of a policy intervention”. I suggest that the Introduction include a section that alludes to this, and why such outcomes could be considered important (e.g., that focusing on mediators can be used as a proxy for actional PA participation outcomes).

 --

 We agree with the inconsistency and the text has been reworded in the following location, text in bold is ‘new’ and has been added for clarity:

 [Introduction page 7 paragraph 1]

 This protocol paper outlines the methodology for seven complementary systematic literature reviews **as part of PEN. Each review** is designed to determine the impact of policy **, either directly or indirectly,** on physical activity outcomes across the different policy domains identified in the “Seven Best Investments” ( ISPAH, 2011).

**Comment 5a:**

 p. 6 Full text review

 Given the importance of implementation to the success of policies, I wonder if it would be worthwhile for the authors to attempt to gather data to indicate whether or not the policy was implemented? Or whether, for example, action plans were developed, and resources allocated to implementation. Given the importance of implementation to policy success, I would see this type of information as critical when evaluating the success of such policies. […]

 --

 While we agree that level of implementation of policies is important, and believe that ‘impact on PA’ will be reflected in the degree of implementation presented, it is beyond the scope of this study to carry out a detailed examination of type of implementation beyond what is reported as evidence of impact. This very important topic may indeed by the subject of a follow up review.

**Comment 5b:**

 […] Also, the inclusion of the validity and reliability of the PA measures should be included in the data extraction.

 --

 Detailed information on any outcome measures used to assess PA will be provided in the data extraction tables and presented in the corresponding reviews. 

**Comment 6:**

 Each of the policy types is very broad, which will make it difficult to identify policy action that is needed to increase PA. For example, a ‘whole-of-school approach’ can includes several different actions. Have the authors considered providing more detail or categorising the type of whole of school approach to facilitate a clearer and more meaningful assessment about the characteristics of effective whole of school policies?

 --

 This links back to Comment 2, the focus of the systematic review is not to evaluate ‘whole of school approach’ but rather the available evidence on impact of direct or indirect policies within specific settings, e.g., the school setting, on PA outcomes.  Therefore, the reviewer is quite correct, we envisage that policy action areas will be broad, e.g., physical education or extra-curricular sport, but the specific policy actions, e.g., require a minimum PE duration or require a PE curriculum may be more defined.  Until the reviews are carried out, the level of evidence is unknown.

**Comment 7:**

 p. 6. Strategy for data synthesis

 I am not clear on what is meant by: “Outcome data extracted will be tabulated to determine the impact on (my emphasis) policy areas and policy actions (policy indicators)”. What is meant here by ‘policy indicators’ for example?

 --

 We agree with the reviewer that this is not clear. In order to improve clarity this sentence has been removed and the third paragraph on page 12 has been restructured as follows, text in bold is ‘new’ and has been added for clarity:

 [Strategy for data synthesis, page 12 paragraph 4]

 “A narrative synthesis will be used to interpret and analyse the data. **The results of the data synthesis will be presented in a table.** **Within this table, a list of short descriptive statements will be compiled based on the policy actions identified in the scientific literature.** Evidence on the effectiveness of these policy actions will then be described using a method **used** by Panter and colleagues (2019) […]”

**Comment 8:**

 I suggest that the method described by Panter and colleagues (2019) be described in the paper (rather than just referring the reader to the paper). It is very important that the reader understands the criteria that is being used to assess the evidence of effectiveness.

 --

 Detail has been added to the second half of the first paragraph on page 12 to describe the coding system inspired by Panter et al. It now reads as follows, text in bold is ‘new’ and has been added for clarity:

 [Strategy for data synthesis, page 12 paragraph 4]

 […] **Specifically,** **when a study presents quantitative evidence about the effectiveness of one policy action a symbol will be assigned next to that particular policy action in the table. There are four categories of symbols reflecting the four possible outcomes:** “significantly positive **evidence**” (+), “significantly negative **evidence**” (-), “no significance test” (?) or “inconclusive” (0).

**Comment 9:**

 I am unclear about how the findings from systematic reviews could be combined with primary research studies. This needs to be further explained.

 --

 This may indeed be challenging, but until we examine the main results and conclusions of any systematic reviews that have reference to policy impact in the targeted domains, we cannot be sure of whether their evidence is strong enough and clearly defined in order to assess impact.  While we agree it may be difficult, in the search strategy we will include these documents in the initial search and depending on what we find include or exclude in the final data synthesis.

**Comment 10:**

 Will there be any attempt to distinguish effectiveness based on the following: school level (primary or secondary school level), gender, level of socio-economic disadvantage?

 --

 Until data extraction is complete, it is difficult to identify any specific stratification variables that would be pertinent to the presentation of the results.  However, the data extraction tables will be devised to take into account any relevant stratification variables for each review as pertinent to the topic and domain.

 As the reviewer’s comment applies to the systematic review corresponding to the school setting only evidence of effectiveness will be presented in a manner that makes it clear which policy actions were shown to be effective at different school levels, across gender and SES where appropriate. The following sentence has been added to the strategy for data synthesis section to clarify this, text in bold is ‘new’ and has been added for clarity.

 [Strategy for data synthesis, page 12 paragraph 3]

**“In addition,  data extraction tables will be designed to distinguish any demographic, environmental or other variables pertinent to synthesising the data. For example, in the schools review , data extraction columns will be included  to reflect evidence of effectiveness stratified by gender, school level (primary, secondary, combined) or socio economic status where appropriate.**
